# Different responses involving Tfh cells delay parasite-specific antibody production in *Trypanosoma cruzi* acute experimental models

**DOI:** 10.3389/fimmu.2025.1487317

**Published:** 2025-04-28

**Authors:** Ana Carolina Leão, Maria Jose Villar, Rakesh Adhikari, Cristina Poveda, Leroy Versteeg, Gregório Almeida, Peter J. Hotez, Maria Elena Bottazzi, Kathryn M. Jones

**Affiliations:** ^1^ Department of Pediatrics, Division of Tropical Medicine, Baylor College of Medicine, Houston, TX, United States; ^2^ Texas Children’s Hospital Center for Vaccine Development, Baylor College of Medicine, Houston, TX, United States; ^3^ Department of Molecular Virology and Microbiology, Baylor College of Medicine, Houston, TX, United States; ^4^ Cell Biology and Immunology Group, Wageningen University & Research, Wageningen, Netherlands; ^5^ Centro de Tecnologia em Vacinas, Universidade Federal de Minas Gerais, Belo Horizonte, Brazil; ^6^ Department of Biology, Baylor University, Waco, TX, United States

**Keywords:** Chagas disease, Tfh, Th1, B cells, antibody, *T. cruzi*

## Abstract

**Introduction:**

Chagas disease (CD), caused by the parasite *Trypanosoma cruzi*, affects millions globally. Despite treatment options in the acute phase, most infections progress to a chronic indeterminate form or develop severe cardiac/gastrointestinal complications. Understanding the immune response is crucial for the development of vaccines and more efficient drugs for the disease control.

**Methods:**

This work investigates the immune response to *T. cruzi* H1 K68 strain infection in female BALB/c and C57BL/6 mice to characterize differences in Tfh and B cell responses that may be involved in the poor parasite-specific antibody production during acute infection. For this, mice were euthanized 14, 28, and 49 days after infection, and splenic T and B cell populations were evaluated by flow cytometry.

**Results:**

BALB/c mice exhibited a strong Th2-biased response with a massive expansion of classic Tfh cells and GC B cells, potentially linked with polyclonal B cell activation and hypergammaglobulinemia, but not with efficient parasite clearance. C57BL/6 mice displayed a Th1-skewed response with a population of "Th1-like Tfh" cells expressing IFN-γ and CXCR5 associated with lower parasite burden and more focused antibody response, including parasitespecific IgG2c during early acute infection.

**Discussion:**

These findings suggest that these mouse models develop different immune responses mediated by Tfh cells, which are crucial for B cell activation and antibody production. The massive expansion of Tfh cells in BALB/c mice might lead to unspecific antibody production due to excessive B cell activation. Conversely, C57BL/6 mice exhibit a "Th1-like Tfh" response lacking classic Tfh cells, potentially explaining their weak parasite-specific antibody production throughout the acute infection. Overall, this study provides for the first time insights into the complex interplay between Tfh cells and antibody production during *T. cruzi* infection, suggesting potential targets for therapeutic intervention in CD.

## Graphical Abstract

Different responses involving Tfh during early *T. cruzi* H1 K68 strain infection. Classic Tfh cell production is extensively induced in BALB/c mice, which accordingly present a massive GC B cell expansion, that can be correlated to hypergammaglobulinemia. C57BL/6 mice presented a “Th1-like Tfh” response with IgG2c parasite-specific antibody production and generation of plasma cells. Created with BioRender.com.

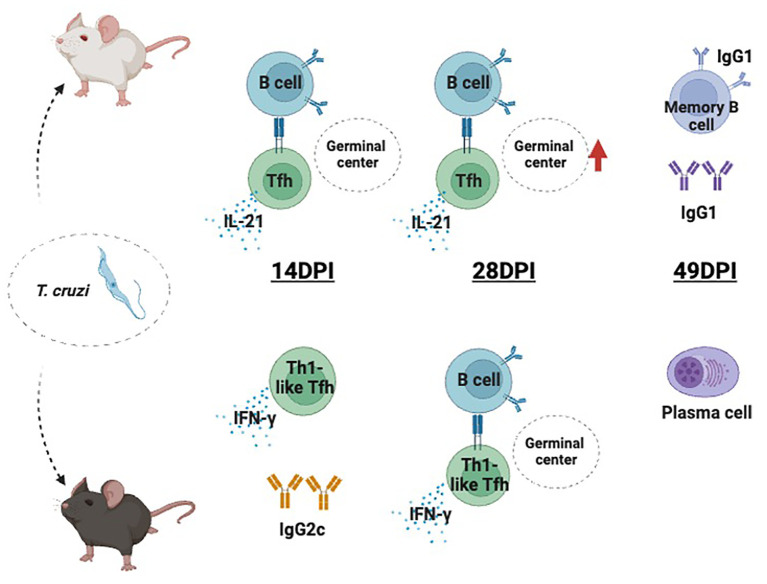

## 1 Introduction

Chagas disease (CD) is a neglected tropical infectious disease caused by the protozoan parasite *Trypanosoma cruzi* (1, 2). About 6–7 million people worldwide are estimated to be infected with *T. cruzi*, and 75 million are at risk of infection (1). CD remains endemic to Latin American countries despite some decreases in infection due to vector control and societal poverty reduction, and urbanization (1, 3). In addition, CD has an increasing global presence derived from human migrations, especially to Southern Europe and Australia (1, 4), and expanding evidence for autochthonous transmission in Texas and elsewhere in the Southern United States (5). Furthermore, there are expanding modes of transmission beyond vector-borne illness, including oral and congenital infections (1, 3, 6).

The clinical outcomes of CD range from asymptomatic to severe chronic cardiovascular or gastrointestinal involvement (7, 8). The acute phase is characterized by high parasitemia, often accompanied by systemic symptoms, such as fever, headache, and diarrhea, among others (9). Occasionally hepatomegaly, splenomegaly, myocarditis, and meningoencephalitis (2, 10). Much less frequently people bitten by a triatomine bug show the characteristic first visible signs, which can be a skin lesion or a purplish swelling of the lids of one eye (inoculation chagoma or unilateral bi-palpebral edema) (1, 2). The acute stage is when the only two available anti-parasitic drugs, Benznidazole and Nifurtimox, exhibit reliable effectiveness (1, 7). However, the symptoms are generally mild, contributing to missed or late diagnosis that leads to the chronic phase of the infection (1, 7). When the parasitemia decreases, most individuals remain asymptomatic or in the indeterminate form, with no clinical symptoms (7, 8). Yet approximately 30% of these patients will develop cardiac and/or gastrointestinal symptoms that may be determinant of the morbidity of the disease (1, 7).

*T. cruzi* infection control depends on both innate and acquired immune mechanisms, including T and B cell responses (11, 12). Macrophages, NK cells, antibodies, CD8^+^ (cytotoxic), and CD4^+^ (helper) T lymphocytes producing high levels of IFN-γ are required to control the parasitemia (13–15). Studies on CD acute experimental models have shown the role of proinflammatory Th1 cytokines, such as IFN-γ and TNF-α, in resistance to *T. cruzi* infection (16, 17), while the Th2 profile is related to disease susceptibility with the production of IL-4 (18, 19). However, in murine experimental studies, over-expression of Th1 immunity can also lead to severe cardiac immunopathology associated with severe inflammation and morbidity. Therefore, effective control of the *T. cruzi* in the mammalian host may rely on balanced Th1/Th2 responses (20). Humoral immunity is also important for parasite control during *T. cruzi* infection; previous studies have shown that B cell depletion increases parasitemia and decreases mice survival in non-lethal infection (15). Further, the adoptive transfer of antibodies from late-stage *T. cruzi*-infected mice to naïve rapidly clears the parasite from the blood (21). Nonetheless, evidence indicates that most B cells are not parasite-specific during early *T. cruzi* infection (19). Also, reports of *T. cruzi* B-cell superantigens, like Tc24 protein, facilitate immune escape by interfering with antibody-mediated responses, eliminating catalytic activity from host innate antibodies (22).

*T. cruzi* has evolved an arsenal of strategies to evade and subvert host immunity, leading to life-long lasting infections. One of these strategies consists of modulating the B cell compartment, attributed to the high variability of parasite surface antigens and the presence of parasite-derived B cell mitogens that cause polyclonal B cell activation and hypergammaglobulinemia (23–26).

Follicular helper T (Tfh) cells are a specialized subset of CD4^+^ T cells that play a crucial role in assisting B cells during the T-dependent germinal center (GC) response, leading to the production of antigen-specific memory B and plasma cells (27). Tfh cells are distinguished from non-Tfh effector cell, like Th1, Th2, or Th17 cell, by their expression of the chemokine receptor CXCR5, costimulatory receptor ICOS, and transcription factor (TF) Bcl6 (27–29). Tfh cells support the GC reaction through their expression of CD40L that engages CD40 on GC B cells, and with the key help of the cytokine IL-21, promote GC B cell proliferation and maintenance and the generation of long-lived Plasma cells (28).

Mouse models have been informative for analyzing immune responses to *T. cruzi* infection. Yet mouse strains show variable disease progression and severity that also differ depending on the parasite strain (29, 30). In general, BALB/c mice are more susceptible to *T. cruzi* infection, compared to relatively resistant C57BL/6 mice, presenting increased parasitemia and mortality given a similar parasite challenge (12, 31). Resistance vs. susceptibility has been linked to C57BL/6 mice Th1 response, in contrast to BALB/c Th2 profile in *T. cruzi* infection, but also *Leishmania*, a related kinetoplastid protozoan species (18, 32). In a study comparing *Leishmania infantum* infection in three murine strains, BALB/c susceptible, C57BL/6 intermediate, and SV/129 resistant, results supported the development of a prevalent Th2-like response in BALB/c, but not in C57BL/6 or SV/129 mice. Also, results showed that both BALB/c and SV/129 presented higher levels of infection-induced splenic Tfh cells than C57BL/6 mice, with parasite-specific antibody significantly higher in SV/129 mice early after infection in comparison with the other two murine strains (33).

CD8^+^ T cell immunity has been extensively studied, given the critical importance of parasite-specific CD8^+^ T cells for host resistance throughout the infection (34, 35). In this work, we compare the immune response to *T. cruzi* H1 K68 strain infection in BALB/c and C57BL/6 mice models to describe for the first time differences in Tfh cell response that may be involved in the lack of parasite-specific antibody production during the initial stages of the infection, which may contribute to relative susceptibility and resistance.

Here, we report the Tfh cells response during *T. cruzi* acute infection by comparing two different mice models. BALB/c and C57BL/6 mice infected with *T. cruzi* H1 K68 strain present distinct responses. While BALB/c has a massive expansion of classic Tfh cells and GC B cells, that could be related to the polyclonal B cell activation and hypergammaglobulinemia. C57BL/6 mice present a “Th1-like Tfh” response (36), whereas the lack of classic Tfh cells may contribute to the poor production of parasite-specific antibodies during acute infection. Tfh different responses in *T. cruzi* infection may help to explain the diversity of CD progressions that range from indeterminate to severe cardiac involvement. A deeper understanding of CD immunopathogenesis is essential for the development of alternatives for treatment and control. Additionally, Tfh immune response presents a key role in vaccine development.

## 2 Materials and methods

### 2.1 Mice and parasite

Female C57BL/6 mice (Jackson Laboratories #000664) and female BALB/c mice (Jackson Laboratories #000651), aged 5–8 weeks, were used for the study. Animal experiments were performed in full compliance with the Guide for the Care and Use of Laboratory Animals, 8th edition (37), under a protocol approved by Baylor College of Medicine’s Institutional Animal Care and Use Committee (IACUC) under assurance number D16-00475. The *T. cruzi* H1 strain (TcI), transfected with the pTRIX2-RE9h plasmid containing the thermostable, red-shifted firefly luciferase gene PpyRE9h (38–40), designated H1 K68 was grown on monolayers of the C2C12 mouse myocyte cell line (ATCC CRL-1772) in RPMI media supplemented with 5% fetal bovine serum and 1X Penicillin/Streptomycin (cRPMI) to propagate tissue culture trypomastigotes (TCT) (41). Culture media containing TCT was collected, parasites were pelleted by centrifugation, washed once with sterile medical grade saline, and resuspended in sterile medical grade saline.

### 2.2 Study design

**Figure 1:**
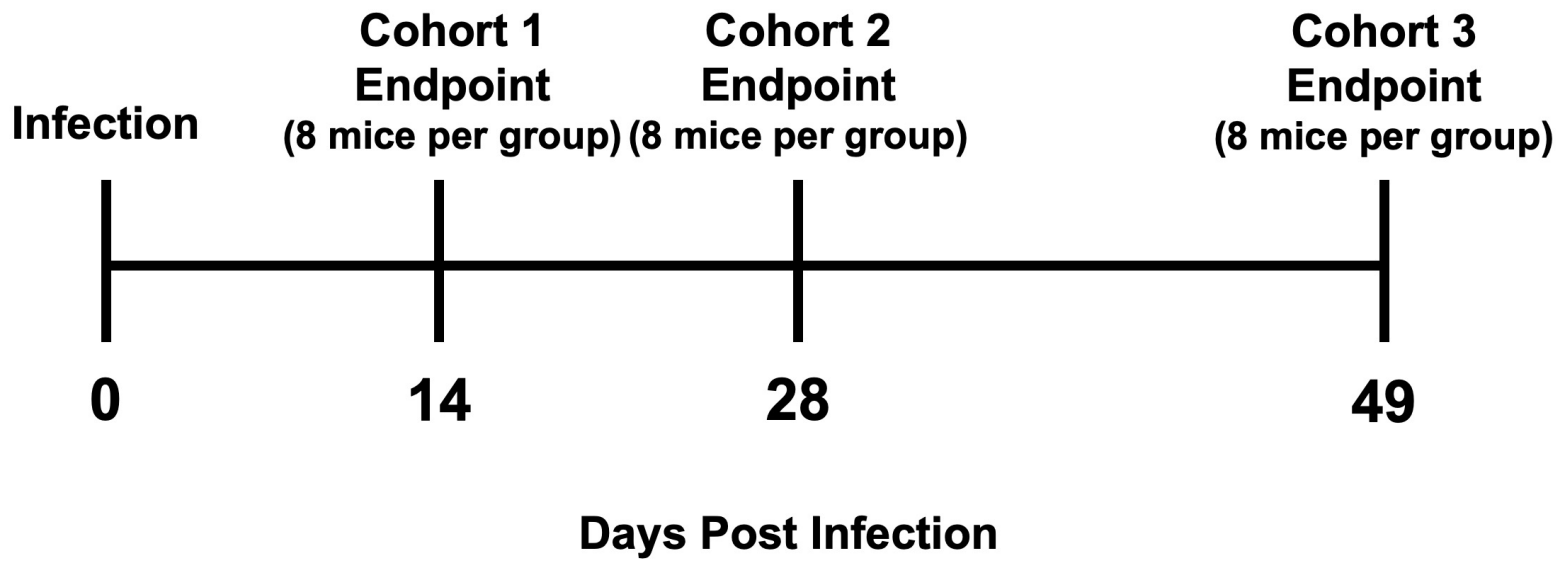
Study design scheme. Timeline with study endpoints.

*T. cruzi* H1 K68 strain (TcI) is a mouse model for Chronic Chagasic cardiomyopathy (CCC) and was selected to ensure the survival of the animals during the study (41, 42). Ninety-six mice (48 BALB/c and 48 C57BL/6) were randomly assigned to form infected or control groups (**Table 1**). The infected groups (24 BALB/and 24 C57BL/6) received by intraperitoneal route 100μL of saline solution containing 5000 trypomastigotes of *T. cruzi* H1 K68, generated in our laboratory (**Table 1**) (41). The control groups (24 BALB/c and 24 C57BL/6) received the same volume of saline solution intraperitoneally (**Table 1**). Blood was collected by tail vein microsampling from all infected mice every week beginning at 7 days post-infection (DPI) to follow parasitemia by quantitative PCR. Mice were monitored daily for morbidity; no clinical signs or mortality were observed during the study. At the study endpoints, 14, 28, and 49 DPI, all mice were humanely euthanized, and hearts, whole blood, femur, and spleens were collected for analysis (**Figure 1**).

**Table 1:** Group scheme.

Group	Size	Mouse Strain	Infection
1	24	BALB/c	None
2	24	BALB/c	5000 T. cruzi H1 K68
3	24	C57BL/6	None
4	24	C57BL/6	5000 T. cruzi H1 K68

### 2.3 QRT-PCR for parasite burden

According to the manufacturer’s guidelines, DNA was extracted from whole blood and frozen heart tissue (20mg) using a PDQeX Nucleic Acid Extractor (MicroGEM). To measure blood and tissue parasite burden, quantitative real-time PCR was performed using TaqMan Fast Advanced master mix (Life Technologies) and oligonucleotides specific for the satellite region of *T. cruzi* nuclear DNA (primers 5′-ASTCGGCTGATCGTTTTCGA-3′ and 5′-AATTCCTCCAAGCAGCGGATA-3′ and probe 5′-6-FAM-CACACACTGGACACCAA-MGB-3′. *T*. *cruzi* data were normalized to glyceraldehyde-3-phosphate dehydrogenase (GAPDH)using the following primers: 5’ CAATGTGTCCGTCGTGGATCT 3’ and 5’ GTCCTCAGTGTAGCCCAAGATG 3’, probe 5’ 6-FAM CGTGCCGCCTGGAGAAACCTGCC MGB 3’; (Life Technologies, CA, USA). After normalization, parasite burden was calculated based on the standard curve of known parasite concentrations. GraphPad Prism software was used to plot parasite equivalents per milliliter of blood over time, and the area under the curve (AUC) was calculated for each animal to determine overall parasitemia. Cardiac parasite burdens were calculated based on a standard curve and expressed as the number of parasites per milligram of tissue (43).

### 2.4 Splenocyte preparation

Spleens were rinsed in sterile 1X PBS, then transferred to a gentleMACS C Tube containing 3 mL of sterile 1X PBS. The tissue was homogenized using a gentleMACS Dissociator (Miltenyi Biotech, Surrey, UK). Red blood cells in the spleen homogenates were subsequently lysed with ACK lysis buffer (Lonza, 10-548E). The lysis solution was diluted 5-fold with RPMI medium supplemented with 10% fetal bovine serum FBS, 1× penicillin-streptomycin (Pen-Strep), and l-glutamine (cRPMI medium). Then splenocytes were pelleted by centrifugation for 5 min at 300 × *g*. Splenocytes were resuspended in 5 mL of cRPMI medium and passed through 40μm strainers (BD Biosciences, 352340). Cells were counted using acridine orange-propidium iodide (AOPI) live/dead dye and a Cellometer Auto 2000 automated cell counter. Then, 1 × 10^6^ live splenocytes were incubated for each sample in a 96-well non-tissue culture plate. For intracellular staining, splenocytes were incubated with 50 ng/mL phorbol 12-myristate 13-acetate (PMA)–500 ng/mL ionomycin, or medium only for 5 h at 37°C in 5% CO_2._ These cells were then analyzed by flow cytometry and Luminex.

### 2.5 Bone marrow preparation

Bone marrow was extracted from the femurs of each mouse using a 25 G x 5/8 in. needle and 10 mL syringe into RPMI-1640 media supplemented with 10% fetal bovine serum FBS, 1× penicillin-streptomycin (Pen-Strep), and l-glutamine (cRPMI medium). The suspension was filtered through 40μm strainers (BD Biosciences, 352340). Cells were counted using acridine orange-propidium iodide (AOPI) live/dead dye and a Cellometer Auto 2000 automated cell counter. Then, 1×10^6^ live cells were incubated for each sample in a 96-well non-tissue culture plate. These cells were then analyzed by flow cytometry.

### 2.6 Flow cytometry analysis

To measure CD4- and CD8- responses, splenocytes were collected 5 hours post restimulation for flow cytometry labeling, washed with PBS, and stained with Live/Dead NIR fixable viability dye, anti-CD3e PE (phycoerythrin)/Dazzle 594, anti-CD4 Alexa Fluor 700, and anti-CD8a peridinin chlorophyll protein (PerCP)-Cy5.5. Tfh was evaluated with anti-CD44 fluorescein isothiocyanate (FITC), anti-CD185 (CXCR5) BV421 Brilliant Violet 421 (BV-421) and anti-CD279 (PD-1) PE (phycoerythrin)-Cy7 (PE-Cy7) (**Supplementary Table S1**). To evaluate intracellular cytokine production, 4.1 μg/ml brefeldin A was added to splenocytes during the 5 h of restimulation. Splenocytes were stained for surface markers as described above, fixed with BD Cytofix/Cytoperm, and permeabilized according to the manufacturer’s instructions. Permeabilized splenocytes were stained with anti-IFN-γ Alexa Fluor 647, anti-TNF Brilliant Violet 605 (BV-605), IL-4 Brilliant Violet 711 (BV-711) and IL-21 phycoerythrin (PE) (**Supplementary Table S1**). To evaluated B cells, splenocytes or bone marrow cells were collected and stained with Live/Dead Aqua fixable viability dye, anti-CD4 FITC, anti-CD8 FITC, anti-Ly-6G/Ly-6C (Gr-1) FITC, anti-F4/80 FITC and anti-CD11c FITC, anti-CD19 BV-421 or BV-711, anti-CD38 PerCP-Cy5.5 or BV-711, anti-CD86 allophycocyanin (APC), anti- CD184 (CXCR4) PE, anti-mouse CD95 (Fas) BV-605, anti-CD138 APC, anti-CD45R (B220) PE-eFluor 610, anti-IgD Alexa Fluor 700, anti-IgM PE-Cy7 and anti-IgG1 PerCP-Cy5.5 (**Supplementary Table S1**). Samples were acquired on an Attune instrument, and at least 100,000 total events in a live gate were analyzed using FlowJo software. Cells were gated on forward and side scatter for lymphocytes, exclusion of viability dye, and singlet populations. T cells (**Supplementary Figure S1**), Tfh cells (**Supplementary Figure S2**), CXCR5^+^CD8^+^ cells (**Supplementary Figure S3**), B cells in the spleen (**Supplementary Figure S4**), and B cells in bone marrow (**Supplementary Figure S5**) were determined based on FMO. Absolute numbers were calculated by multiplying the frequency of live cells from the population of interest by the total number of cells collected from the bone marrow. Data were plotted using GraphPad Prism software.

### 2.7 Multiplex analysis of cytokines by Luminex

Cell-free supernatant from re-stimulated splenocytes was collected after 5 h and frozen at -80°C until Luminex analysis. Cytokines levels of IL-2, IL-4, IL-6, IL-10, IFN-γ, and TNF-α in supernatants were measured by Luminex-based assay using the Milliplex MAP Mouse kit (Millipore) as previously described (44).

### 2.8 ELISA parasite-specific IgM, IgG, IgG1, and IgG2a/c

Indirect ELISAs were conducted to measure *T. cruzi* parasite antibody titers from serum. 96-well NUNC ELISA plates were coated overnight at 4°C with 2 μg/mL of *T. cruzi* H1 K68 strain parasite lysate (45) in 1x coating buffer (KPL). The coating buffer was discarded the following day, and the plates were blocked with assay buffer (0.1% BSA/1X PBS/0.05%Tween 20) for 2 h at room temperature. Mouse serum was serially diluted two-fold in assay buffer, starting at a 1:200 dilution. For the negative control, pooled naïve mouse sera were also diluted to 1:200. ELISA plates were washed using a BioTek 405 TS plate washer and PBST (1X PBS/0.05%Tween 20). Diluted mouse serum and the controls were added to the washed ELISA plates in duplicate at 100 μL/well and incubated for 2 h at room temperature. Following incubation, the plates were washed four times. Then, 100 μL/well of the appropriate secondary antibodies were added: 1:6000 diluted goat anti-mouse IgM HRP (Lifespan Bioscience), goat anti-mouse IgG1 HRP (Lifespan Bioscience), goat anti-mouse IgG2a HRP (Lifespan Bioscience), or 1:40000 diluted goat anti-mouse Ig2c HRP (Lifespan Bioscience). After 1 h of incubation at room temperature, the ELISA plates were washed five times. Then, 100 μL of TMB substrate (KPL) was added to each well and incubated for 15 minutes. The reaction was stopped by adding 100 μL/well 1M HCl, and the absorbance at 450 nm was measured using an Epoch 2 spectrophotometer (Biotek). Duplicate values of the measured O.D. at 450 nm were averaged for data analysis. The titer cutoff value was calculated as follows: titer cutoff = average negative control + 3 x standard deviation of the negative control. The titer was determined for each mouse serum sample by taking the corresponding dilution factor of the highest dilution with an average O.D. value above the titer cutoff. If a sample did not show an average O.D. value above the titer cutoff at 1:200, an arbitrary titer value of 67 was assigned (baseline).

### 2.9 Statistical analysis

Statistical analysis was performed using GraphPad Prism software version 9.3.1 for Windows (GraphPad Software, San Diego, California, USA). Significance was calculated by the Normality test followed by the parametric Unpaired t-test or nonparametric Mann-Whitney test. P values ≤0.05 were considered significant. The figures’ P-values ≤0.05, ≤0.01, ≤0.001, and ≤0.0001 are represented as one, two, three, and four symbol characters, respectively.

## 3 Results

### 3.1 BALB/c but not C57BL/6 mice infected with *T. cruzi* present a classic Tfh cell response

**Figure 2:**
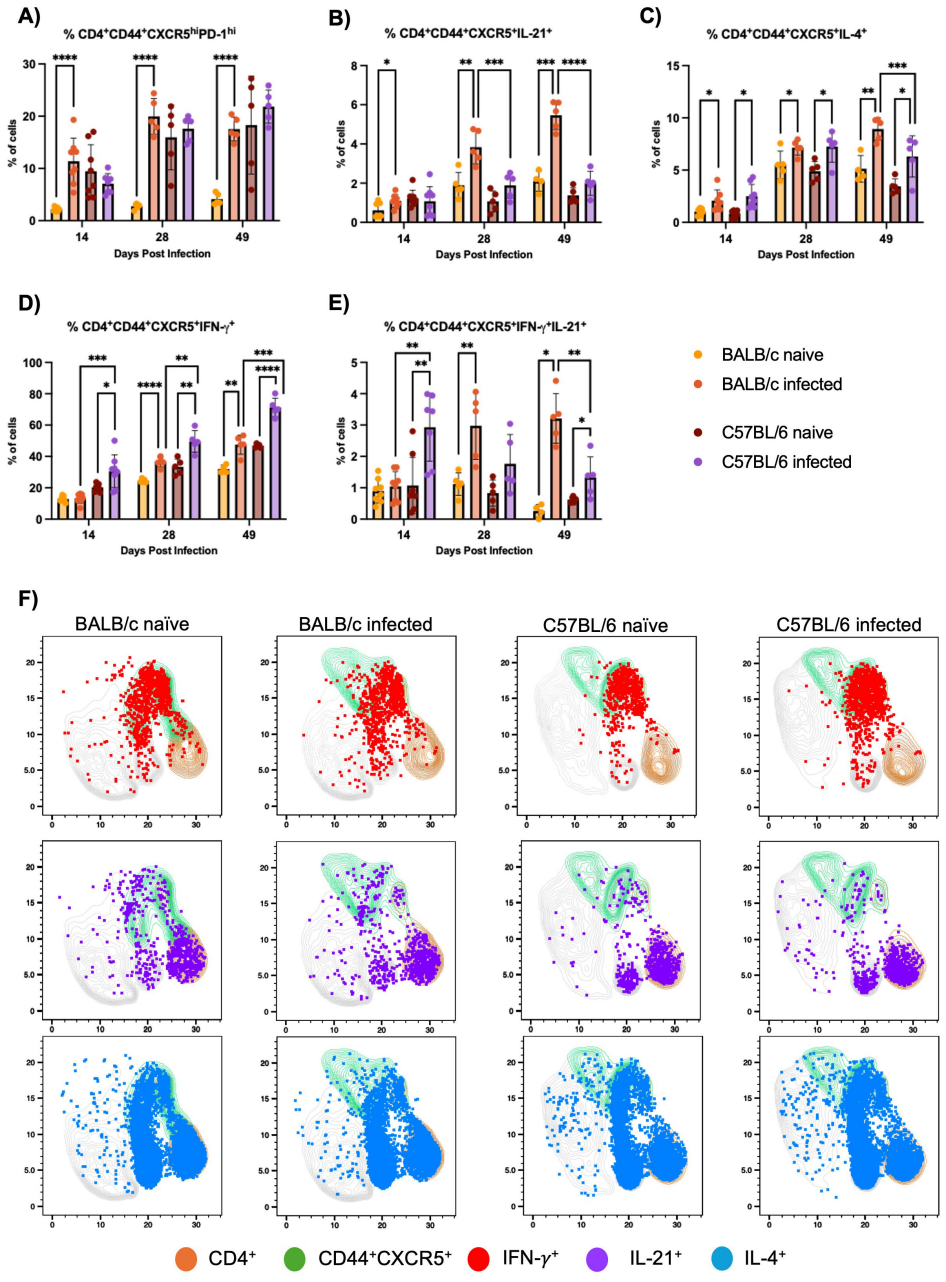
Tfh cell response evaluation. Flow cytometry analysis of splenocytes restimulated with PMA/I for 5 h from BALB/c and C57BL/6 mice naïve or infected with 5000 trypomastigotes of H1 K68 strain of *T. cruzi*. **(A)** Analysis of CD4^+^CD44^+^CXCR5^hi^PD-1^hi^ **(B)** CD4^+^CD44^+^CXCR5^+^IL-21^+^ **(C)** CD4^+^CD44^+^CXCR5^+^IL-4^+^ **(D)** CD4^+^CD44^+^CXCR5^+^IFN-γ^+^ cells and **(E)** UMAP projection of IFN-γ, IL-21 and IL-4 production by CD4^+^ and CD4^+^CD44^+^CXCR5^+^T lymphocytes compartments at 14 DPI. Each point represents an individual mouse; 14 DPI n = 8, 28 DPI n=5 and 49 DPI n=5. Significance was calculated by the Normality test followed by the parametric Unpaired t-test or nonparametric Mann-Whitney test. P-values of ≤0.05, ≤0.01, ≤0.001, and ≤0.0001 are represented as one (*), two (**), three (***), and four (****) symbol characters, respectively.

BALB/c mice exhibit a classical Tfh cell response, including expansion of IL-21-producing Tfh cells, compared to C57BL/6 mice during H1 K68 *T. cruzi* infection. Tfh cell response was evaluated by flow cytometry. Results showed that BALB/c infected mice compared to naïve show an increase in CD4^+^CD44^+^CXCR5^hi^PD-1^+^ Tfh cells at 14, 28, and 49 DPI, which is not present in the C57BL/6 model (**Figure 2A**). In the same direction, when IL-21 Tfh-producing cells are evaluated, an expansion of CD4^+^CD44^+^CXCR5^+^IL-21^+^ cells are observed in BALB/c infected mice compared to naïve in all time points but not in C57BL/6 (**Figure 2B**). In addition, these cells are expanded in BALB/c infected mice compared to C57BL/6 infected mice at 28 and 49 DPI (**Figure 2B**). Otherwise, when CD4^+^CD44^+^CXCR5^+^IFN-γ^+^ cells are evaluated, we observe an expansion in C57BL/6 infected mice compared to naïve at 14, 28, and 49 DPI, while in BALB/c mice this increase is present only at 28 and 49 DPI (**Figure 2D**). Analyzing the infected group from both mouse models, there is an expansion of these cells in C57BL/6 compared to BALB/c mice in all time points (**Figure 2D**). Interestingly, evaluating CD4^+^CD44^+^CXCR5^+^ cells that are both IFN-γ^+^ and IL-21^+^, C57BL/6 infected mice show an increase of these cells compared to naïve and to BALB/c infected mice at 14 DPI (**Figure 2E**). However, at 28 DPI this population is only expanded in BALB/c infected mice compared to naïve (**Figure 2E**). Yet at 49 DPI, this polyfunctional cells are expanded in both infected mouse models compared to naïve, and in BALB/c infected mice compared to C57BL/6 infected mice (**Figure 2E**).

Finally, the evaluation of CD4^+^CD44^+^CXCR5^+^IL-4^+^ cells showed that in the infected group from both models, these cells are expanded at 14, 28, and 49 DPI compared to naïve (**Figure 2C**). But at 49DPI BALB/c infected mice also present an expansion of these cells compared to C57BL/6 infected group (**Figure 2C**). A Uniform Manifold Approximation and Projection (UMAP) was generated to analyze cytokine production by CD4^+^CD44^+^CXCR5^+^ T cells at 14 DPI (**Figure 2F**). Results showed that most IFN-γ^+^ cells overlap with CD44^+^CXCR5^+^, contrarily to IL-21^+^ cells. In contrast, IL-4^+^ cells are spread in CD4^+^ regions that are CD44^+^CXCR5^+^ and not positive for these markers (**Figure 2F**).

### 3.2 Expansion of CD8^+^CXCR5^+^ cells in mice infected with *T. cruzi*

**Figure 3:**
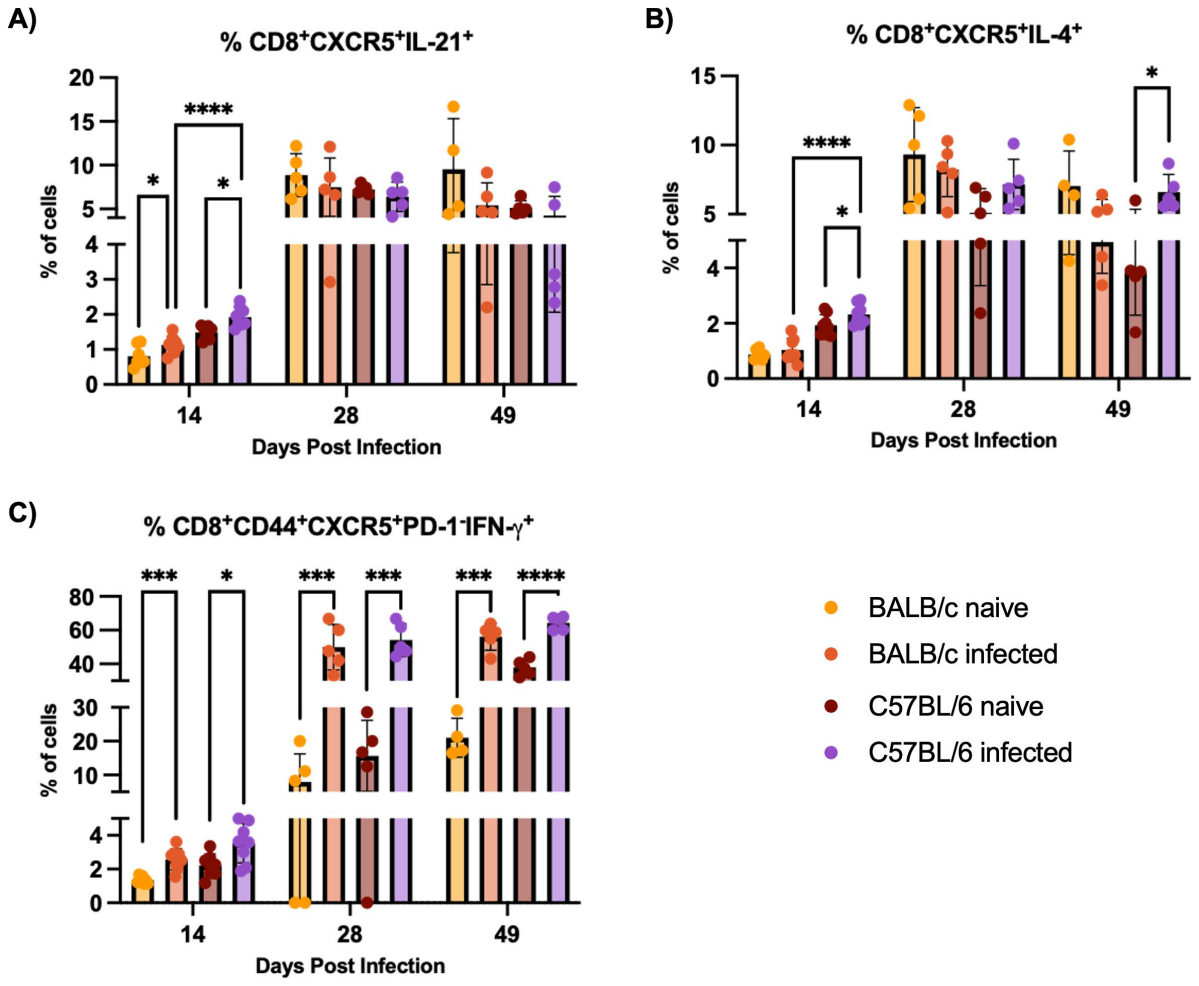
CD8^+^CXCR5^+^ cells response evaluation. Flow cytometry analysis of splenocytes restimulated with PMA/I for 5 h from BALB/c and C57BL/6 mice naïve or infected with 5000 trypomastigotes of H1 K68 strain of *T. cruzi*. **(A)** Analysis of CD8^+^CXCR5^+^IL-21^+^ **(B)** CD8^+^CXCR5^+^IL-4^+^ and **(C)** CD8^+^CD44^+^CXCR5^+^PD-1^-^IFN-γ^+^ cells. Each point represents an individual infected mouse: 14 DPI n = 8, 28 DPI n=5, and 49 DPI n=5. Significance was calculated by the Normality test followed by the parametric Unpaired t-test or nonparametric Mann-Whitney test. P-values of ≤0.05, ≤0.001, and ≤0.0001 are represented as one (*), three (***) and four (****) symbol characters, respectively.

CD8^+^CXCR5^+^ T cells, including IL-21-producing subsets, are expanded in both BALB/c and C57BL/6 mice during H1 K68 *T. cruzi* infection, with C57BL/6 mice showing a more pronounced expansion at 14 DPI. Recent work in different models and diseases has identified unique subsets of CD8^+^ T cells expressing the chemokine receptor CXCR5, which directs these cells into secondary lymphoid follicles (47). Comparing H1 K68 *T. cruzi* infection using BALB/c and C57BL/6 mice, flow cytometry results showed that IL-21 CD8^+^CXCR5^+^ producing cells, that have been described as antibody-enhancement (B cell “helper”) subset (48, 49), are increased in infected mice compared to naïve in both models only at 14 DPI (**Figure 3A**). CD8^+^CXCR5^+^IL-4^+^ population is expanded exclusively in C57BL/6 infected mice compared to naïve at 14 and 49 DPI (**Figure 3B**). IL-21 or IL-4 CD8^+^CXCR5^+^ producing cells are also expanded in C57BL/6 infected mice compared to BALB/c infected mice at 14DPI (**Figures 3A, B**). Another CD8^+^CXCR5^+^ subset that has been reported as antibody-suppressor (50, 51) and described as CD8^+^CD44^+^CXCR5^+^PD-1^-^CXCR5^+^IFN-γ^+^ cells was also evaluated. Results showed that these cells are increased in the infected group compared to naïve in both models at 14, 28, and 49 DPI (**Figure 3C**).

### 3.3 Characterization of the B cell response in BALB/c and C57BL/6 mice infected with *T. cruzi*

**Figure 4:**
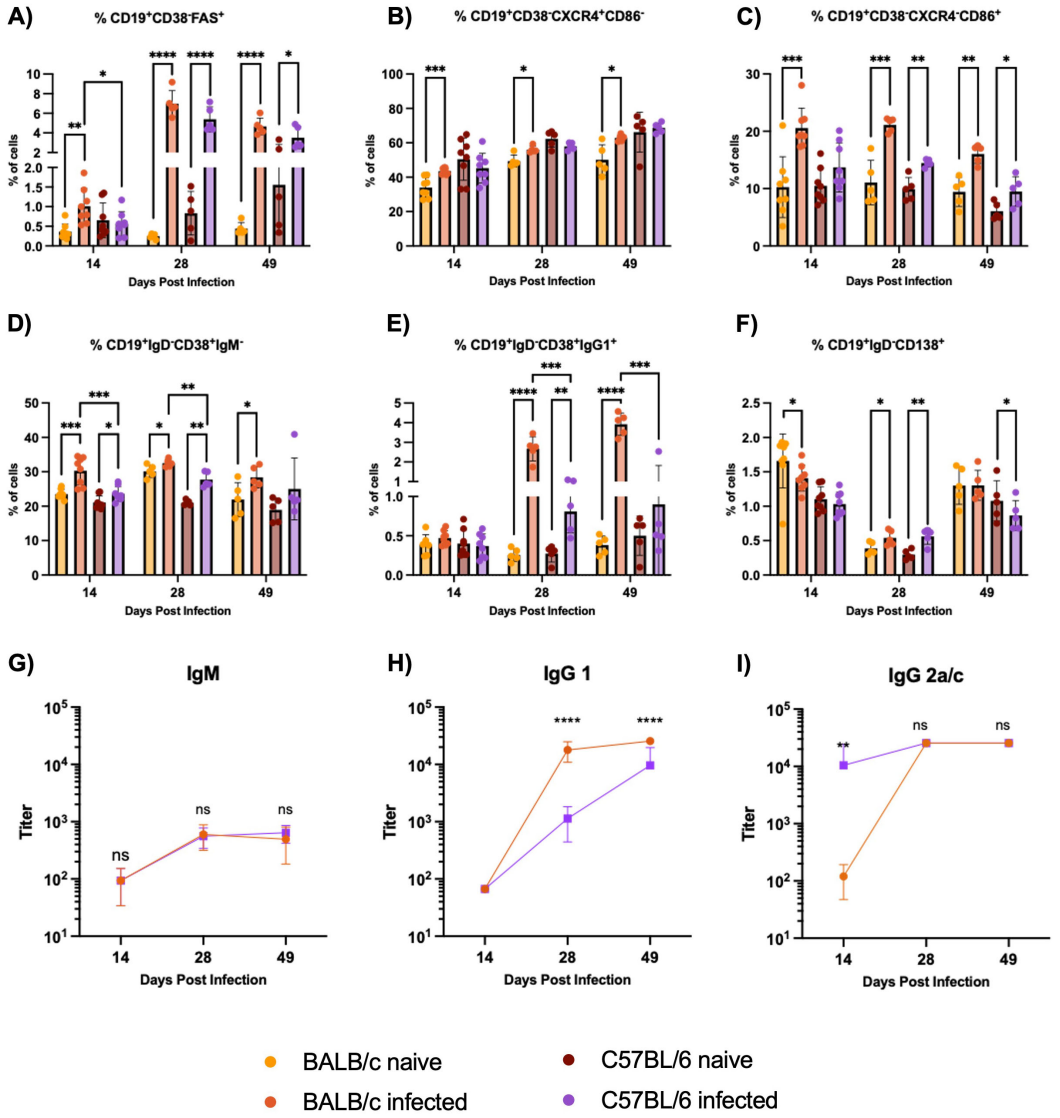
B cell immune response evaluation in the spleens. Flow cytometry analysis of B cells from non-stimulated splenocytes from BALB/c and C57BL/6 mice naïve or infected with 5000 trypomastigotes of H1 K68 strain of *T. cruzi*. **(A)** GC B cells CD19^+^CD38^-^FAS^+^ **(B)** Dark zone GC B cells CD19^+^CD38^-^FAS^+^CXCR4^+^CD86^-^ **(C)** Light zone GC B cells CD19^+^CD38^-^FAS^+^CXCR4^-^CD86^+^ **(D)** Isotype switched memory B cells (MBC) CD19^+^CD38^+^ IgM^-^ **(E)** IgG1^+^MBC CD19^+^CD38^+^IgG1^+^ **(F)** Antibody secreting cells (ASC) CD19^+^IgD^-^CD138^+^ **(G)** IgM *T. cruzi*-specific antibody titers **(H)** IgG1 *T. cruzi*-specific antibody titers **(I)** IgG2a/c *T. cruzi*-specific antibody titers. For the flow cytometry results, each point represents an individual mouse: 14 DPI n = 8, 28 DPI n=5, and 49 DPI n=5. Each point represents the average of 5 infected mice sera for the ELISA results. Significance was calculated by the Normality test followed by the parametric Unpaired t-test or nonparametric Mann-Whitney test. P-values of ≤0.05, ≤0.01, ≤0.001, and ≤0.0001 are represented as one (*), two (**), three (***), and four (****) symbol characters, respectively.

BALB/c mice exhibit an earlier and stronger expansion of germinal center B cells compared to C57BL/6 mice during H1 K68 *T. cruzi* infection, with BALB/c mice producing higher levels of parasite-specific IgG1. To start the B cell immune response characterization, germinal center (GC) B cells were evaluated by flow cytometry. Results showed that in BALB/c infected mice, the expansion of CD19^+^CD38^-^FAS^+^ GC B cells starts at 14 DPI, massively expands at 28 DPI, and continues at 49 DPI (**Figure 4A**). In C57BL/6 mice, the expansion of GC B cells in infected mice compared to naïve only happens at 28 DPI and continues at 49 DPI (**Figure 4A**). Also, this population is expanded in BALB/c infected mice compared to C57BL/6 infected mice at 14 DPI (**Figure 4A**). When the dark zone (DZ), CD19^+^CD38^-^FAS^+^ CXCR4^+^CD86^-^ cells, and the light zone (LZ), CD19^+^CD38^-^FAS^+^ CXCR4^-^CD86^+^ cells, components of the GC B cells were evaluated, we observe a significant increase in both populations in BALB/c infected mice at 14, 28 and 49 DPI compared to naïve (**Figures 4B,C**). Meanwhile, in C57BL/6 infected mice, only LZ GC B cells increase starting at 28 DPI and continuing at 49 DPI (**Figures 4B, C**).

Following the B cell response characterization, memory B cells (MBC) and antibody-secreting cells (ASC) were evaluated by flow cytometry. Comparing naïve and infected groups, we observe an increase in the isotype switched C19^+^CD38^+^IgM^-^ MBC cells in BALB/c infected mice at 14, 28, and 49 DPI. In C57BL/6 infected mice, this expansion is present only at 14 and 28 DPI (**Figure 4D**). In addition, this population is expanded in BALB/c infected mice compared to C57BL/6 infected mice at 14 and 28 DPI (**Figure 4D**). IgG1^+^MBC were also analyzed, results showed that these cells are highly expanded in BALB/c infected mice compared to naïve at 28 and 49 DPI, while in C57BL/6 infected mice, this population is increased only at 28 DPI (**Figure 4E**). These cells are also expanded in BALB/c infected mice compared to C57BL/6 infected mice at 28 and 49 DPI (**Figure 4E**). The percentage of CD19^+^IgD^-^CD138^+^ ASC is reduced in BALB/c infected mice compared to the naïve at 14 DPI (**Figure 4F**). However, at 28 DPI, these cells are expanded in BALB/c and C57BL/6 infected mice compared to the naïve groups (**Figure 4F**). Additionally, IgM, IgG1, and IgG2a for BALB/c mice, and IgG2c for C57BL/6 mice, *T. cruzi*-specific antibody titers were analyzed by ELISA. Results showed no significant differences in IgM production between BALB/c and C57BL/6 mice (**Figure 4G**). Also, higher titers of parasite-specific IgG1 are produced in BALB/c compared to C57BL/6 infected mice at 28 and 49 DPI (**Figure 4H**). However, C57BL/6 Ig2c titers were higher than BALB/c Ig2a at 14 DPI, and no differences were observed at 28 and 49 DPI (**Figure 4I**).

### 3.4 *T. cruzi* infection induces higher levels of antibody secreting cells (ASC) in C57BL/6 mice bone marrow

**Figure 5:**
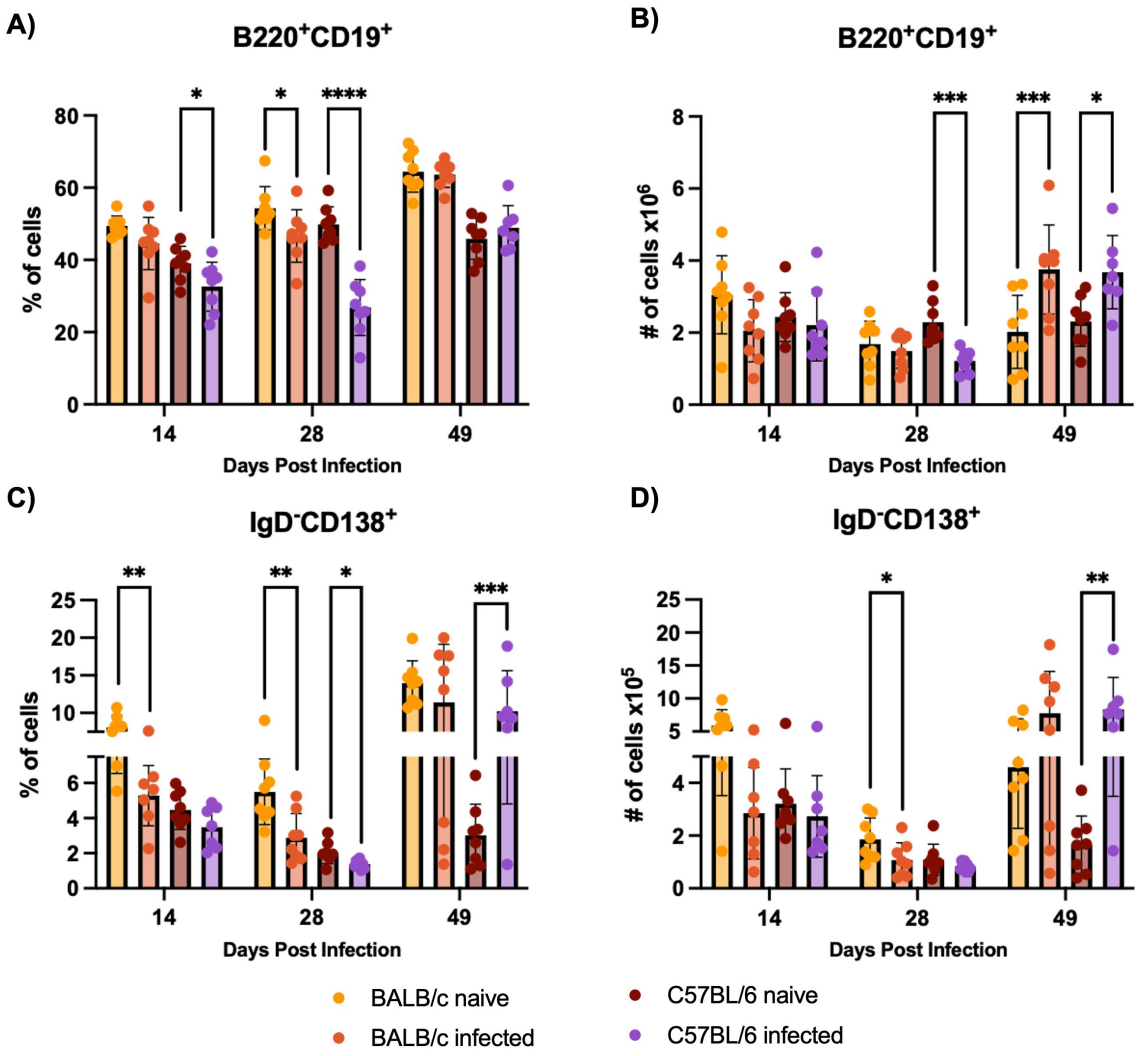
B cell immune response evaluation in the bone marrow. Flow cytometry analysis of bone marrow cells from BALB/c and C57BL/6 mice naïve or infected with 5000 trypomastigotes of H1 K68 strain of *T. cruzi*. **(A)** Percentage of B220^+^CD19^+^ Total B cells **(B)** Number of B220^+^CD19^+^ Total B cells **(C)** Percentage of CD19^+^IgD^-^CD138^+^ Antibody secreting cells (ASC) **(D)** Number of CD19^+^IgD^-^CD138^+^ ASC. Each point represents an individual mouse: 14 DPI n = 8, 28 DPI n=5, and 49 DPI n=5. Significance was calculated by the Normality test followed by the parametric Unpaired t-test or nonparametric Mann-Whitney test. P-values of ≤0.05, ≤0.01, ≤0.001, and ≤0.0001 are represented as one (*), two (**), three (***), and four (****) symbol characters, respectively.

In response to H1 K68 *T. cruzi* infection, both BALB/c and C57BL/6 mice show a reduction in total B cell and antibody-secreting cell populations in the bone marrow at earlier time points (14 and 28 DPI). But by 49 DPI, there is an increase in B cell numbers in both strains, with C57BL/6 mice showing a rebound in ASC population. B cell immune response to H1 K68 *T. cruzi* infection was also evaluated in the bone marrow. Flow cytometry analysis showed that the percentage of B220^+^CD19^+^ total B cells decreases when we compare C57BL/6 naïve to infected mice at 14 and 28 DPI (**Figure 5A**). In addition, we observe a reduction in the number of these cells at 28 DPI (**Figure 5B**). BALB/c infected mice present a reduction in the frequency of total B cells at 28 DPI (**Figure 5A**). At 49 DPI, in both models, the infected mice have an increment in the number of these cells compared to the naïve (**Figure 5B**). ASC were also evaluated, the percentage of CD19^+^IgD^-^CD138^+^ cells decreased when we compared BALB/c naïve to infected mice at 14 and 28 DPI (**Figure 5C**). C57BL/6 infected mice present a reduction in ASC frequency at 28 DPI, yet an increment at 49 DPI compared to the naïve (**Figure 5C**). The number of this population decreased in BALB/c infected mice compared to naïve at 28 DPI and increased in C57BL/6 infected mice compared to the control at 49 DPI (**Figure 5D**).

### 3.5 Parasite burden is increased in BALB/c compared to C57BL/6 during early acute phase

**Figure 6:**
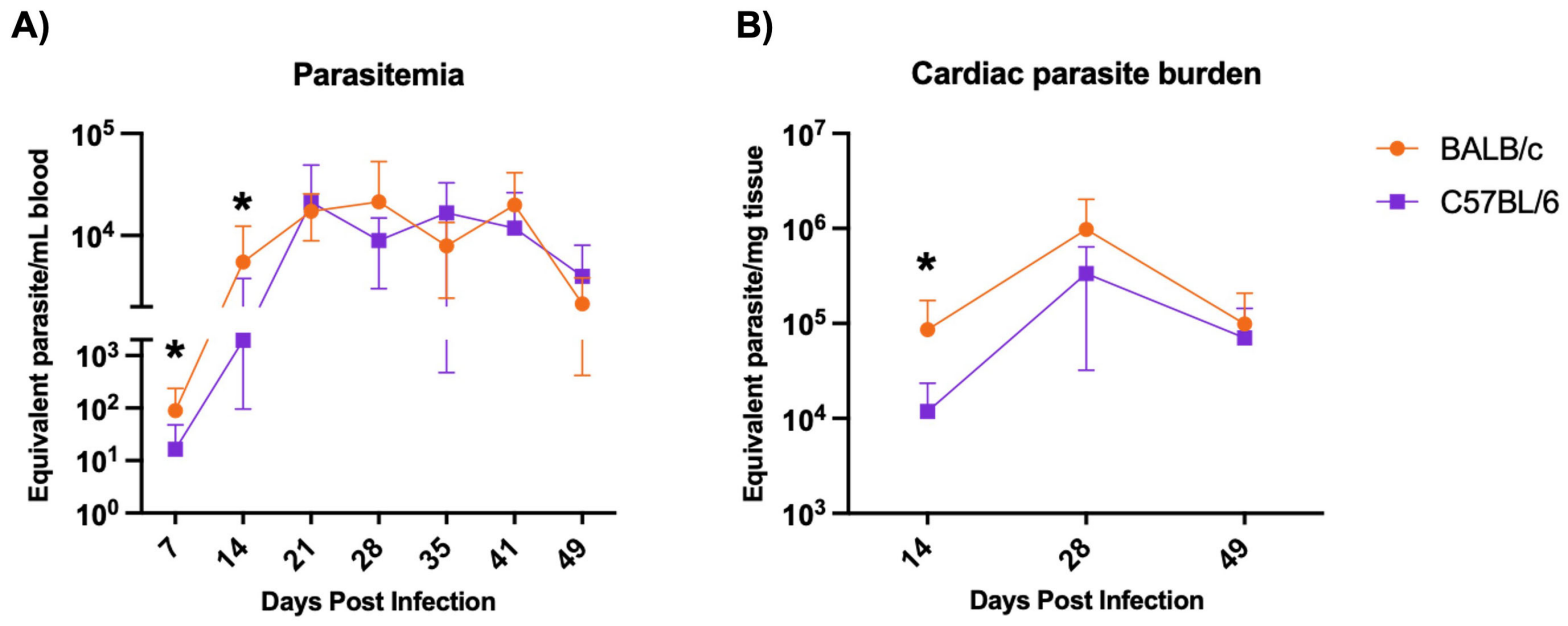
Parasite burden evaluation. Mice were infected with 5000 bft *T.cruzi* strain H1 – K68. **(A)** Infection was monitored by measuring parasitemia weekly. Each point represents mean values: 7 DPI n=24, 14 DPI n=24, 21 DPI n=16, 28 DPI n=16, 35 DPI n=8, 41 DPI n=8 and 49 DPI n=8. **(B)** Cardiac parasite burden was quantified by quantitative real-time PCR. Each point represents mean values n=8. Significance was calculated using the nonparametric Mann-Whitney test and the area under the curve (AUC). P-values of ≤0.05 are represented as one (*) symbol character. AUC from 7 and 14 DPI comparing BALB/c and C57BL/6 p value = 0.0172.

BALB/c mice exhibit significantly higher parasitemia at 7 and 14 DPI and higher cardiac parasite burden at 28 DPI compared to C57BL/6 mice during H1 K68 *T. cruzi* infection. To compare *T. cruzi* infection between the two mouse models, BALB/c and C57BL/6 mice were infected with *T. cruzi* H1 K68, as described above. Parasites in blood were monitored weekly from 7 to 49 DPI (**Figure 6A**). Cardiac parasite burden was evaluated at the endpoints 14, 28, and 49 DPI (**Figure 6B**). BALB/c mice had significantly increased parasitemia at 7 and 14 DPI and cardiac parasite burden at 28 DPI compared to the C57BL/6 mice model (**Figure 6A, B**).

### 3.6 Secreted cytokines multiplex analysis and T cell response evaluation confirm C57BL/6 Th1 and BALB/c Th2 profiles

**Figure 7:**
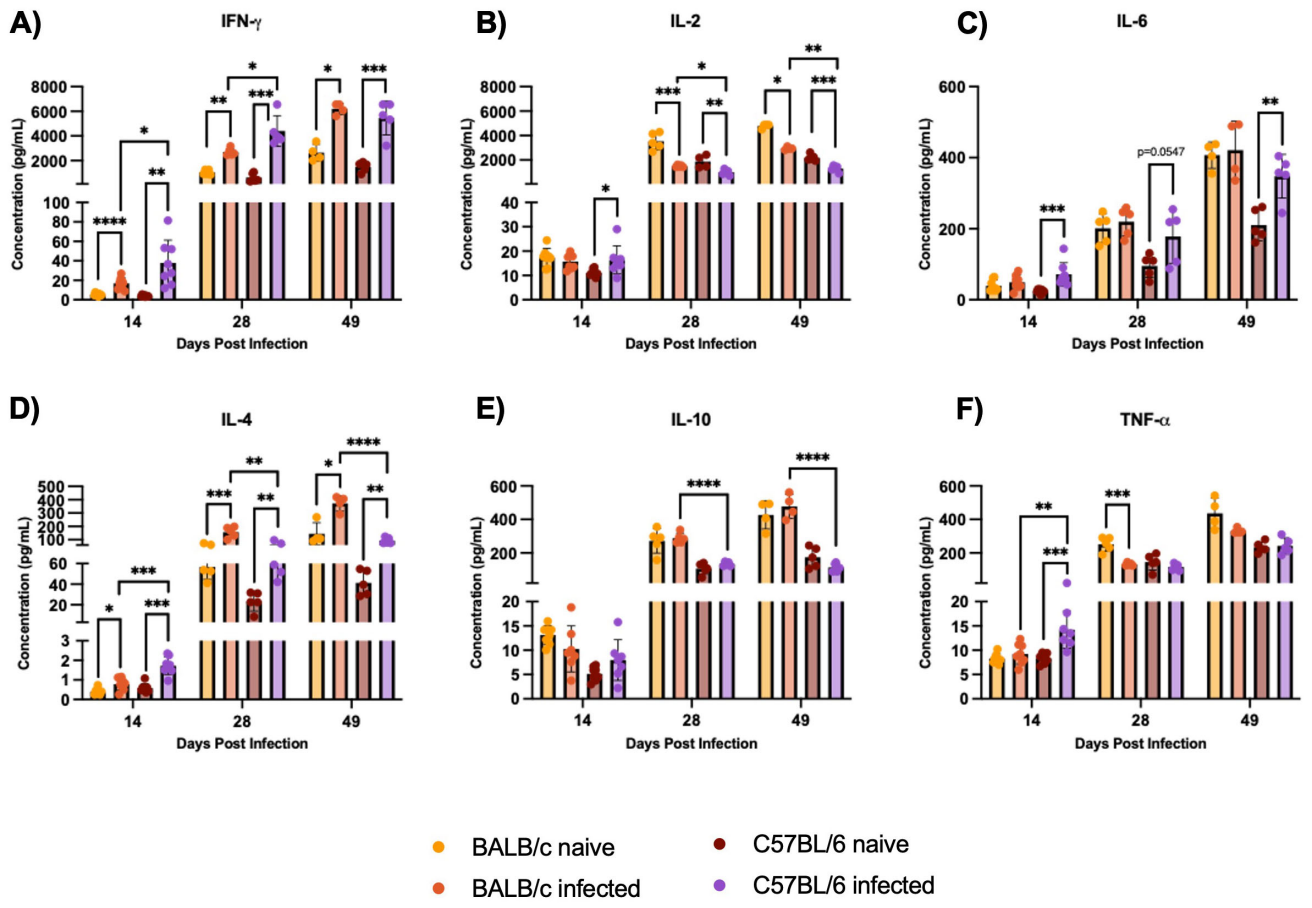
Multiplex analysis of secreted cytokines by Luminex. Splenocytes were restimulated with PMA/I for 5h. The multiplex analysis measured secreted cytokines from these splenocytes. **(A)** IFN-γ **(B)** IL-2 **(C)** IL-6 **(D)** IL-4 **(E)** IL-10 **(F)** TNF-α. Each point represents an individual mouse: 14 DPI n = 8, 28 DPI n=5, and 49 DPI n=5. Significance was calculated by the Normality test followed by the parametric Unpaired t-test or nonparametric Mann-Whitney test. P-values of ≤0.05, ≤0.01, ≤0.001, and ≤0.0001 are represented as one (*), two (**), three (***), and four (****) symbol characters, respectively.

C57BL/6 mice exhibit a Th1 skewed immune response, with higher levels of IFN-γ and TNF-α while BALB/c mice show a Th2 response, characterized by higher IL-4 production, during H1 K68 *T. cruzi* infection. The characterization of the immune response by multiplex analysis of secreted cytokines was also performed. At 14 DPI, higher levels of IFN-γ and TNF- α were generated in C57BL/6 compared to BALB/c-infected mice, that continues at 28 DPI for IFN-γ (**Figures 7A, F**). Still, at 14 DPI, we have a significant increase of IL-4 in C57BL/6 compared to BALB/c, whereas, at 28 and 49 DPI, the concentration of IL-4 is higher in BALB/c than in C57BL/6 infected mice (**Figure 7D**). At 28 and 49 DPI, the levels of IL-2 and IL-10 are also higher in BALB/c compared to C57BL/6 infected mice (**Figures 7B, E**). Additionally, comparing infected to naïve mice, in C57BL/6 there are increased levels of IL-2 at 14 DPI and reduced levels at 28 and 49 DPI (**Figure 7B**). IL-6 is increased in C57BL/6 infected mice throughout the acute infection (**Figure 7C**). In BALB/c and C57BL/6 mice, IFN-γ and IL-4 are increased in the infected group compared to naïve throughout the acute infection (**Figures 7A, D**). While IL-2 is reduced at 28 and 49 DPI, and TNF-α only in BALB/c at 28 DPI (**Figures 7B, F**). Cytokine-producing CD4^+^ and CD8^+^ T-cells were also evaluated by flow cytometry to compare BALB/c and C57BL/6 mice responses to H1 K68 *T. cruzi* infection (**Supplementary Figure S6**).

## 4 Discussion

Induction of an appropriate immune response to *T. cruzi* during acute infection determines whether parasite burdens and tissue damage are quickly controlled to minimize organ dysfunction or if excessive inflammation leads to progressive damage and clinical disease. Here, we performed a thorough immune evaluation focused on elucidating the early kinetics of Tfh cell and B cell development, contributing to effective parasite control. Tfh cells are distinguishable from naive or other effector T cells by expression of the chemokine receptor CXCR5, which directs Tfh cells to the B cell follicle, and of the exhaustion marker PD-1 (28, 52). Poor Tfh response is associated with a defective GC reaction, while the overabundance can lead to pathogenic autoantibody production and autoimmune disease (53). We confirmed that our BALB/c infection model is relatively susceptible, with higher parasite burdens and a Th2-biased immune response, compared to the relatively resistant C57BL/6 infection model, with lower parasite burdens and a Th1-biased immune response. These data are in accordance with other acute CD (as well as other parasitic kinetoplastid) models that link proinflammatory Th1 cytokines like IFN-γ to resistance and Th2 response with IL-4 production to susceptibility to *T. cruzi* infection (17, 19).

To better understand the molecular and cellular mechanisms of this established observation, we showed that BALB/c and C57BL/6 mice infected with *T. cruzi* H1 K68 strain present different responses regarding Tfh cells. BALB/c infected mice exhibited massive expansion of CD4^+^CD44^+^CXCR5^hi^PD-1^+^ Tfh cells and increased IL-21-producing Tfh cells during acute infection. “Hybrid Th1/Tfh” or “Th1-like Tfh” have been used to describe any IFN-γ^+^ CD4 T cell also expressing IL-21 and/or CXCR5, functional markers of Tfh in Malaria models (36, 54). Here, we describe for the first time the production of CD4^+^CD44^+^CXCR5^+^IFN-γ^+^ cells during *T. cruzi* infection, with a high production of these cells in C57BL/6 infected mice. C57BL/6 mouse groups also showed frequencies of CD4^+^CD44^+^CXCR5^hi^PD-1^+^ Tfh cells as high as BALB/c infected mice. However, there is no difference between naive and infected C57BL/6 mice, suggesting that this high frequency is not caused by the infection. Further investigation is necessary to evaluate the specificity of this response to *T. cruzi* infection. In addition, the same is not observed when we analyzed IL-21 Tfh-producing cells, what corroborates that C57BL/6 mouse infected with *T. cruzi* H1 K68 do not present a classical Tfh cell response.

Recently, CXCR5^+^CD8 T cells have been described in several pathogenic conditions with varied functional capacity (55). In murine and human studies, antibody-enhancing CXCR5^+^CD8^+^ T cells have been reported to express IL-21 (48, 49). Interestingly, at 14 DPI C57BL/6 infected mice show higher frequencies of IL-21 CD8^+^CXCR5^+^ producing cells than BALB/c infected mice. What as well could be related to some protection observed in C57BL/6 mice. The role of IL-4-producing CD8^+^ T cells still hasn’t been well characterized; however, in the CD experimental model, the production of IFN-γ by CD8^+^ T cells in the absence of IL-4 has been linked to cardiac tissue damage (56). When CD8^+^CXCR5^+^IL-4^+^ is evaluated, we observe a significant increase in these cells in C57BL/6 mice compared to BALB/c. It remains to be investigated whether IL-4 produced by CD8^+^CXCR5^+^ would exert the same protective role in *T. cruzi* infection.

In contrast, antibody-suppressor CXCR5^+^CD8^+^ T cells have been identified in transplantation models as another subset by the absence of the co-inhibitory molecule PD-1, the lack of IL-21 production, and the expression of IFN-γ (47, 50). CD8^+^CD44^+^CXCR5^+^PD-1^-^IFN-γ^+^ evaluation showed that there is an expansion of this population in infected mice compared to the naïve in both models. Alloantibody suppression was described as mediated, partially, by CD8-mediated clearance of antibody-producing B cells through both FasL and perforin mechanisms (51). This cytotoxic clearance has been shown to be antigen-specific, as CD8^+^ T Ab-supp cells do not kill naïve or third-party primed IgG^+^ B cells *in vitro* or *in vivo* (47, 51). The Fas receptor/Fas ligand (FasR/FasL) pathway in *T. cruzi* infection has been described as one of the main mechanisms involved in B cell death, participating in the selectively elimination of IgG^+^ B cells reactive to parasite but not self-antigens (57, 58).

During the early stages of infection, *T. cruzi* causes polyclonal B cell activation, leading to hypergammaglobulinemia, yet most of the antibodies produced are not parasite-specific and unable to control infection (59–61). Correlated to the massive expansion of Tfh cells in BALB/c mice infected with H1 K68 *T. cruzi* strain, when we evaluated GC B cells, a vast expansion of this population was also observed in BALB/c mice. Despite not presenting a Tfh classic response, C57BL/6 mice also expanded GC B cells compared to naïve, but only at 28 and 49 DPI. Previous work has shown that C57BL/6 mice infected with *T. cruzi* Y strain presented lower polyclonal B cell activation than BALB/c mice, suggesting that polyclonal activation in *T. cruzi* infection highly depends on the host strain (30). This difference was associated with the Th1-focused C57BL/6 immune response, in contrast to the Th2-focused response developed by BALB/c mice (30). Nevertheless, it has been described that IL-4, predominantly secreted by Tfh and Th2 cells after antigen encounter, is crucial for the survival of GC B cells (62). Further, it has been suggested in *Plasmodium* spp. infection that the hybrid Th1/Tfh population producing IFN-γ, IL-21, and IL-10 are likely to concurrently provide cellular protection and limit the large humoral response that leads to hypergammaglobulinemia (36).

When the dark zone (DZ) and the light zone (LZ) GC B cells were evaluated, C57BL/6 infected mice only presented an expansion of the LZ compared to naïve mice, while BALB/c infected mice presented an expansion on both compartments. B cells in the DZ proliferate and undergo somatic hypermutation, producing cells that express antibodies that differ in their ability to bind antigen (28). Mutant GC B cells subsequently migrate to the LZ, where they test their newly mutated receptors and receive help from cognate Tfh cells, leading to positive selection of cells expressing higher-affinity antibodies (28). The polyclonal activation of the B cell compartment could restrict the size of the niche needed for optimal development of antigen-specific lymphocytes by increasing competition for activation and survival signals in the lymphoid tissues, resulting in a delayed parasite-specific antibody response (61, 63).

MBC and ASC were evaluated to assess the impact of the different Tfh responses in B cell development. IgG1^+^MBC is greatly expanded in BALB/c compared to C57BL/6 mice. Additionally, elevated titers of parasite-specific IgG1 are also found in sera from BALB/c-infected mice. IL-4 has been linked to class-switch recombination to IgG1 in mice; remarkably, IL-4 from Tfh cells rather than classical Th2 cells (62). In C57BL/6 mice, IgG2c and IgG2a in the BALB/c are more efficient than other subclasses in neutralizing viruses (64). C57BL/6 *T. cruzi* parasite-specific IgG2c antibody titers were higher than BALB/c IgG2a at 14 DPI. Although the GC was canonically thought to be the site of class-switch recombination, recent work suggests that switching occurs primarily before complete GC maturation in pre-GC B cells (28).

Another strategy to manipulate the B cell response by *T. cruzi* is the reduction in B cells in the bone marrow during the infection, enhancing B cell apoptosis and affecting B cell migration to the periphery (65, 66). After H1 K68 *T. cruzi* strain, B220^+^CD19^+^ total B cell decreases when we compare naïve to infected mice, but ASC increases at 49 DPI in the C57BL/6 model.

Studies have shown the role of cytokines that are signaled through signal transducer and activator of transcription 3 (STAT3), like IL-6 and IL-21, in promoting the Tfh cell phenotype (53, 62). STAT3 expression in CD4^+^ T cells is required for their differentiation into Tfh cells and promotion of GC B cell development and virus-specific antibody responses (53). In a lymphocytic choriomeningitis virus (LCMV) acute infection model, it was demonstrated that STAT3 downmodulates type I interferon (IFN) signaling, as STAT3-deficient Tfh cells display a marked increase in Th1 cell-associated and interferon-inducible transcripts (53). Also, antibody blockade of the IFNαβ receptor promoted Tfh cell differentiation in wild-type mice and mice containing STAT3-deficient CD4^+^ T cells (53). In a mouse model of Chronic Chagasic Cardiomyopathy (CCC), mice infected with *T. cruzi* H1 strain and then treated with TTI-101, a small molecule inhibitor of STAT3, showed that STAT3 inhibition eliminated cardiac fibrosis but increased cardiac inflammation. Also, upregulated cardiac gene expression of STAT1, IL-6 and Type I and Type II IFN responses (67).

Collectively, in this work, results suggest that different responses involving Tfh lead to poor and delayed antibody production during early *T. cruzi* H1 K68 strain infection. Classic Tfh cell production is extensively induced in BALB/c mice, which accordingly present a massive GC B cell expansion, that can be correlated to polyclonal B cell activation and hypergammaglobulinemia, generating unspecific antibodies. Contrarily, C57BL/6 mice presented a “Th1-like Tfh” response that restrained this hypergammaglobulinemia and could be connected to the IgG2c parasite-specific antibody production. This response may have contributed to the better performance of the C57BL/6 model, with less parasites in the blood and heart at 14 DPI. Concurrent, results suggest that H1 K68 *T. cruzi* infection doesn’t induce the expansion of classic Tfh cells in the C57BL/6 mice model, and it is possible that the lack of this population compromises a stronger specific antibody response during the infection.

Further investigation is needed to assess if the differences in Tfh response could be related to a higher activation of STAT3 over type I IFNs in BALB/c mice, with the opposite happening in the C57BL/6 model. As well to understand the role of Tfh and “Th1-like Tfh” response in *T. cruzi* infection and the impact of these cells in the generation of long-lived plasma cells in the bone marrow. Study evaluating circulating Tfh cell subsets (cTfh) from adult patients with different clinical forms of chronic Chagas disease showed phenotypic changes between asymptomatic patients and patients with chagasic dilated cardiomyopathy, suggesting that dysregulation of Tfh cells might contribute to Chagas disease progression (9). Moreover, clarifying the role of CD8^+^CXCR5^+^ cells in *T. cruzi* infection and elucidating whether a balanced Th1 and Tfh response would improve host resistance are also needed. The primary function of Tfh cells is to provide protection from infectious diseases, facilitating antibody responses to viral, bacterial, parasite, and fungal infections (46). Potentially, a better understanding of the Tfh response during *T. cruzi* infection could aid in the development of immunotherapies that generate an earlier and stronger parasite-specific antibody response to control disease progression.

## Data Availability

The original contributions presented in the study are included in the article/**Supplementary Material**. Further inquiries can be directed to the corresponding author.
