# Supplementary material for: Different responses involving Tfh cells delay parasite-specific antibody production in Trypanosoma cruzi acute experimental models
Source: Front Immunol. 2025 Apr 28;16:1487317. doi: 10.3389/fimmu.2025.1487317 (PMC12066522; doi:10.3389/fimmu.2025.1487317)
Supplement: Supplementary file 1 [file DataSheet1.pdf]

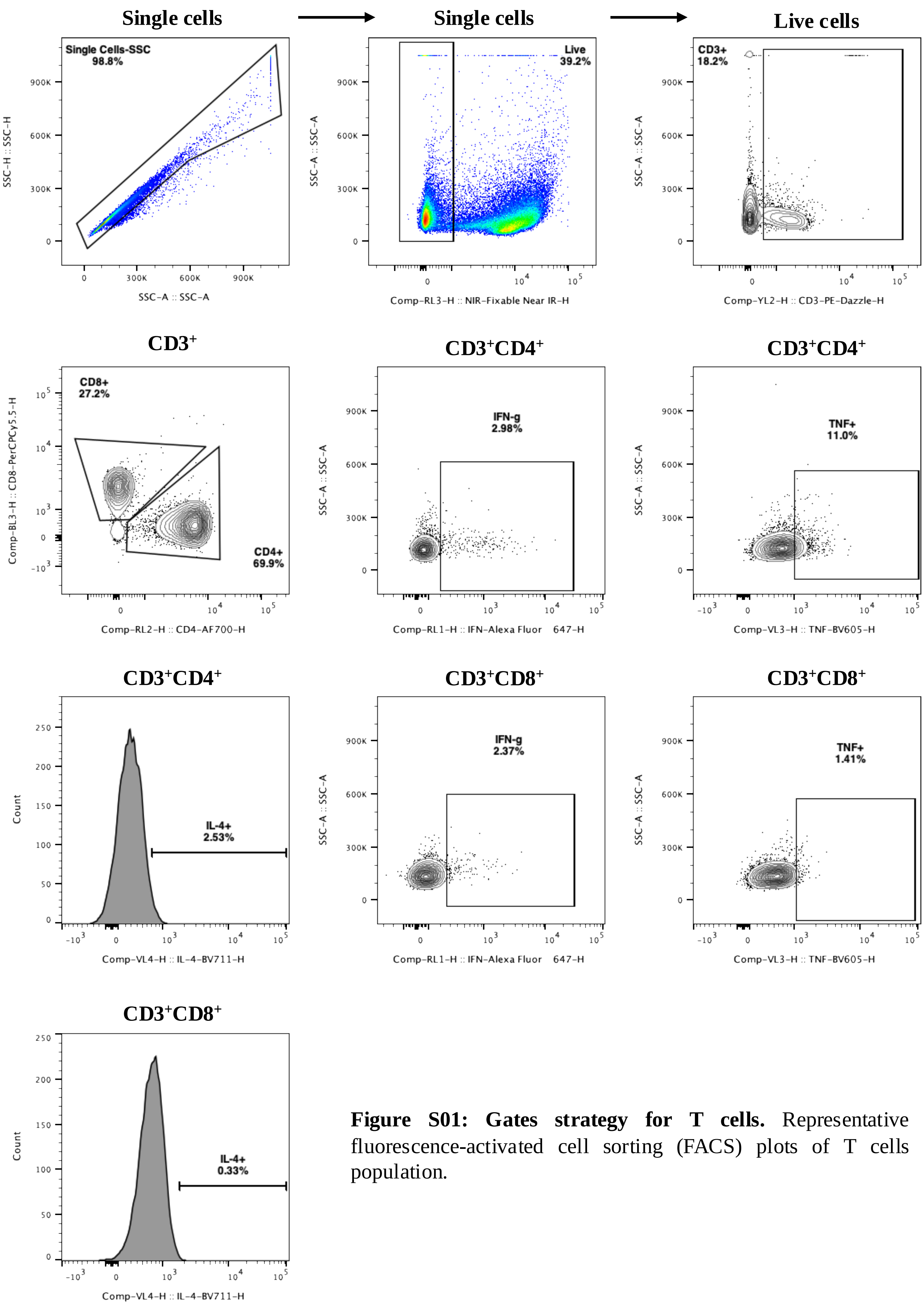

Figure S01: Gates strategy for T cells. Representative fluorescence-activated cell sorting (FACS) plots of T cells population.

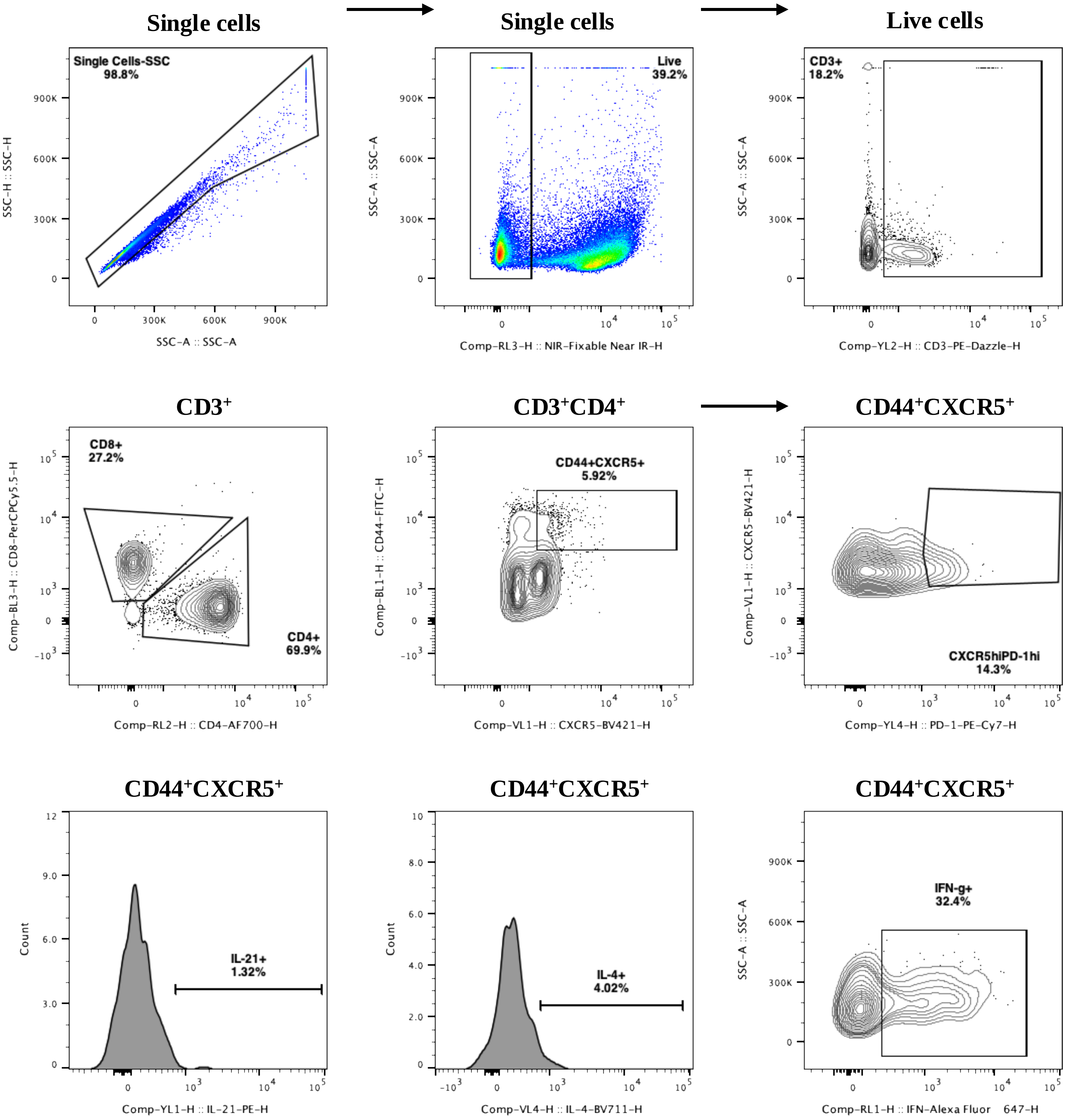

Figure S02: Gates strategy for Tfh cells. Representative fluorescence-activated cell sorting (FACS) plots of Tfh cells population.

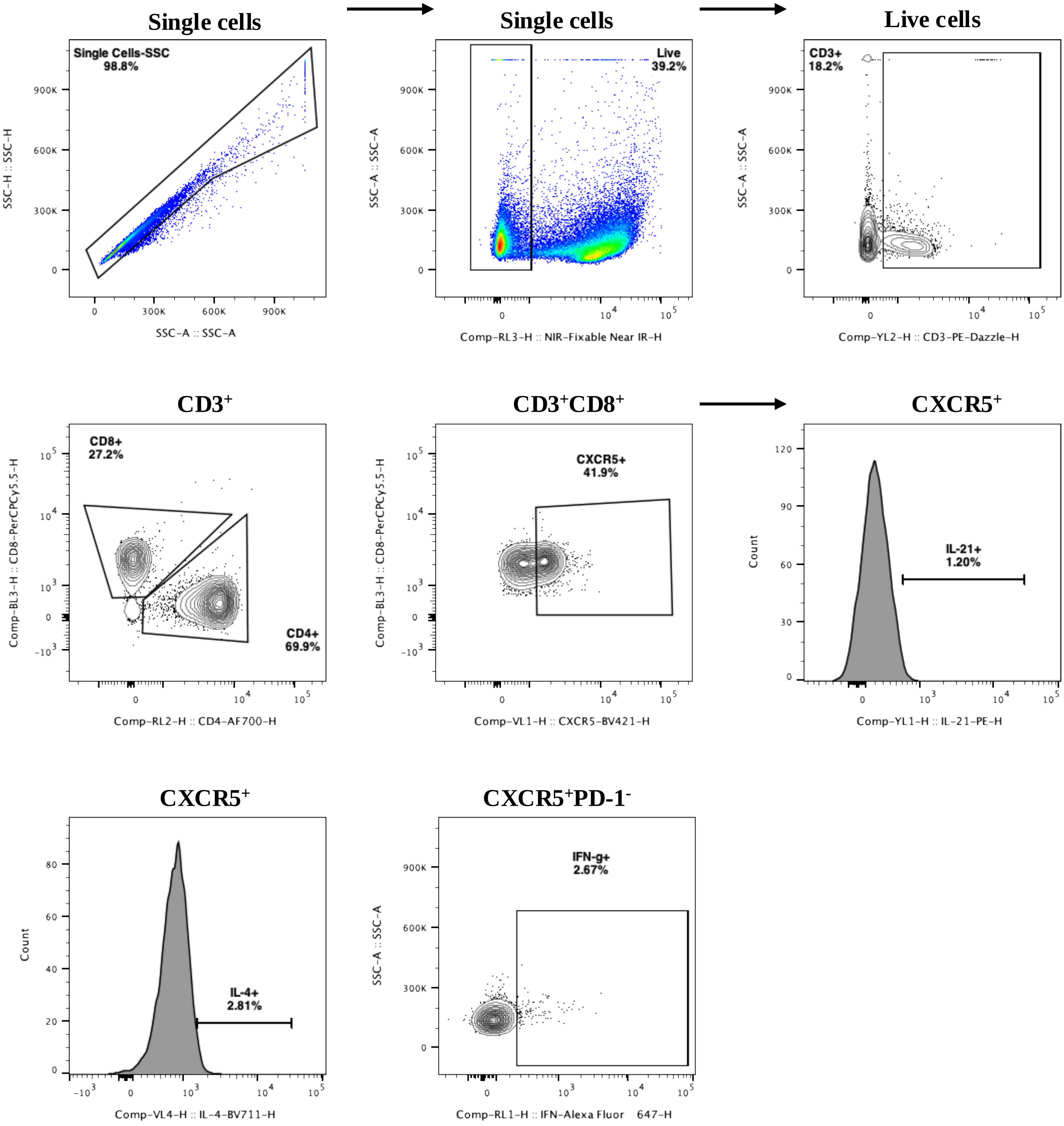

Figure S03: Gates strategy for CXCR5$^+$CD8$^+$ cells. Representative fluorescence-activated cell sorting (FACS) plots of CXCR5$^+$CD8$^+$ cells population.

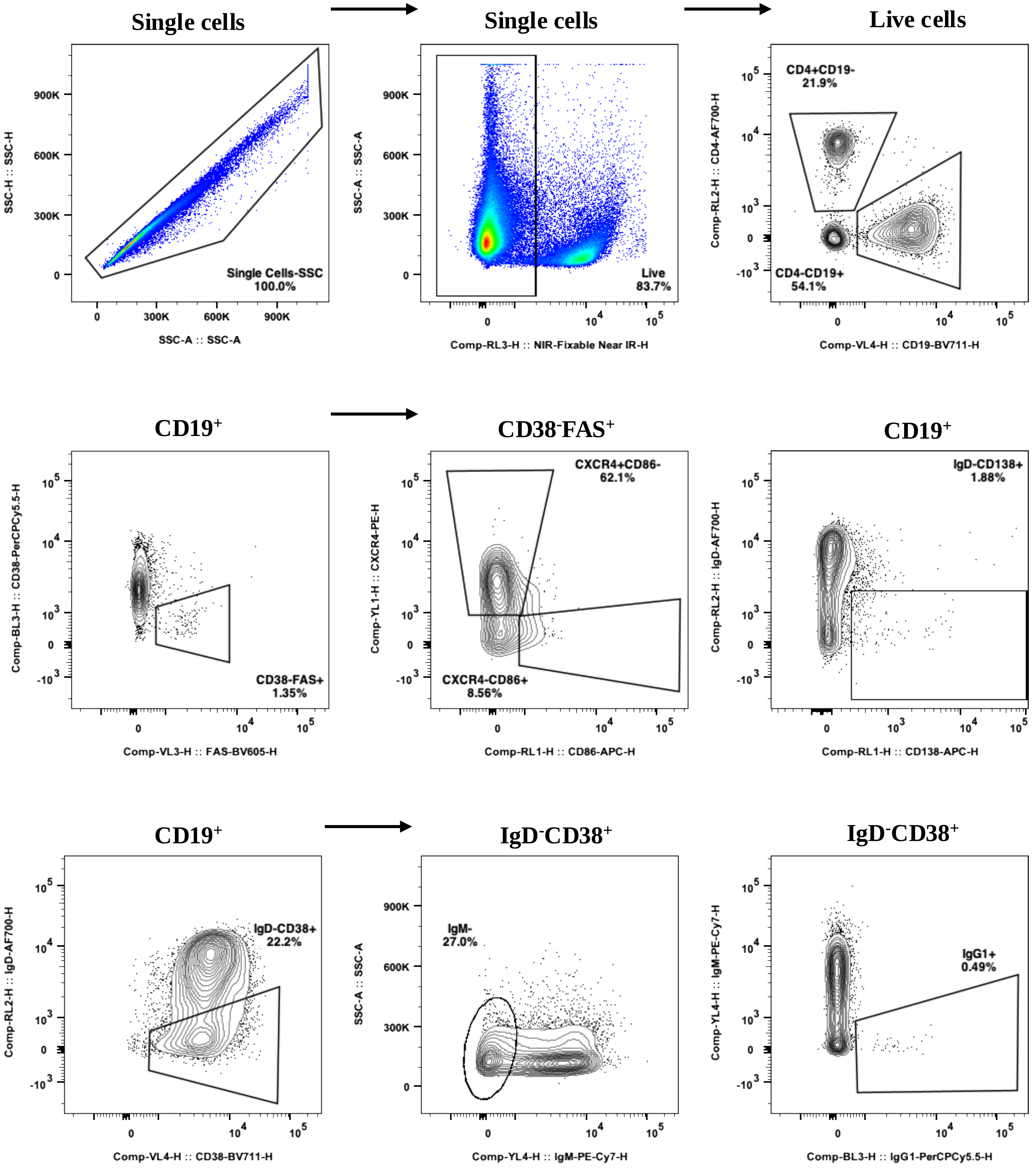

Figure S04: Gates strategy for B cells in spleen. Representative fluorescence-activated cell sorting (FACS) plots of B cells population.

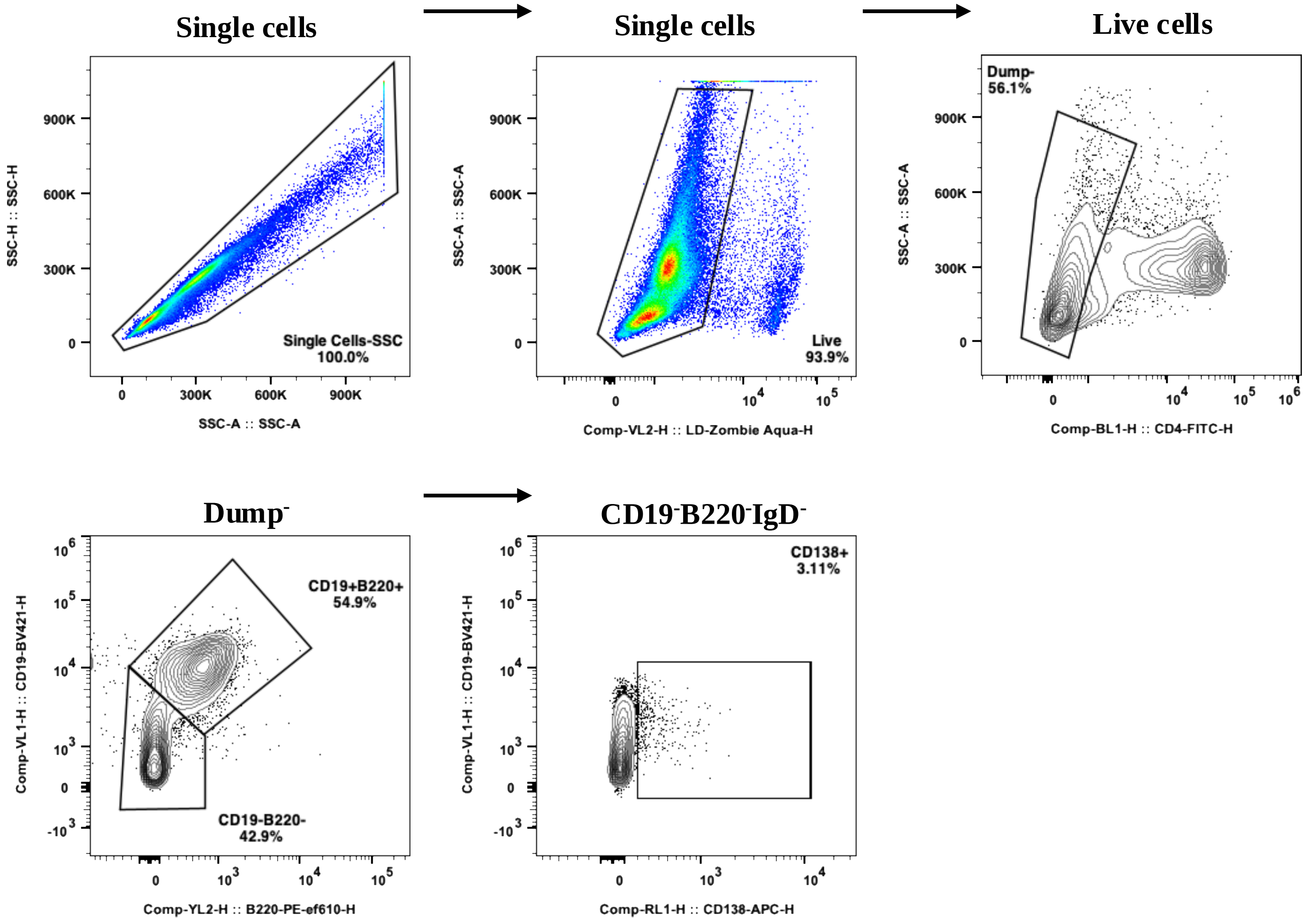

Figure S05: Gates strategy for B cells in bone marrow. Representative fluorescence-activated cell sorting (FACS) plots of B cells population in spleen.

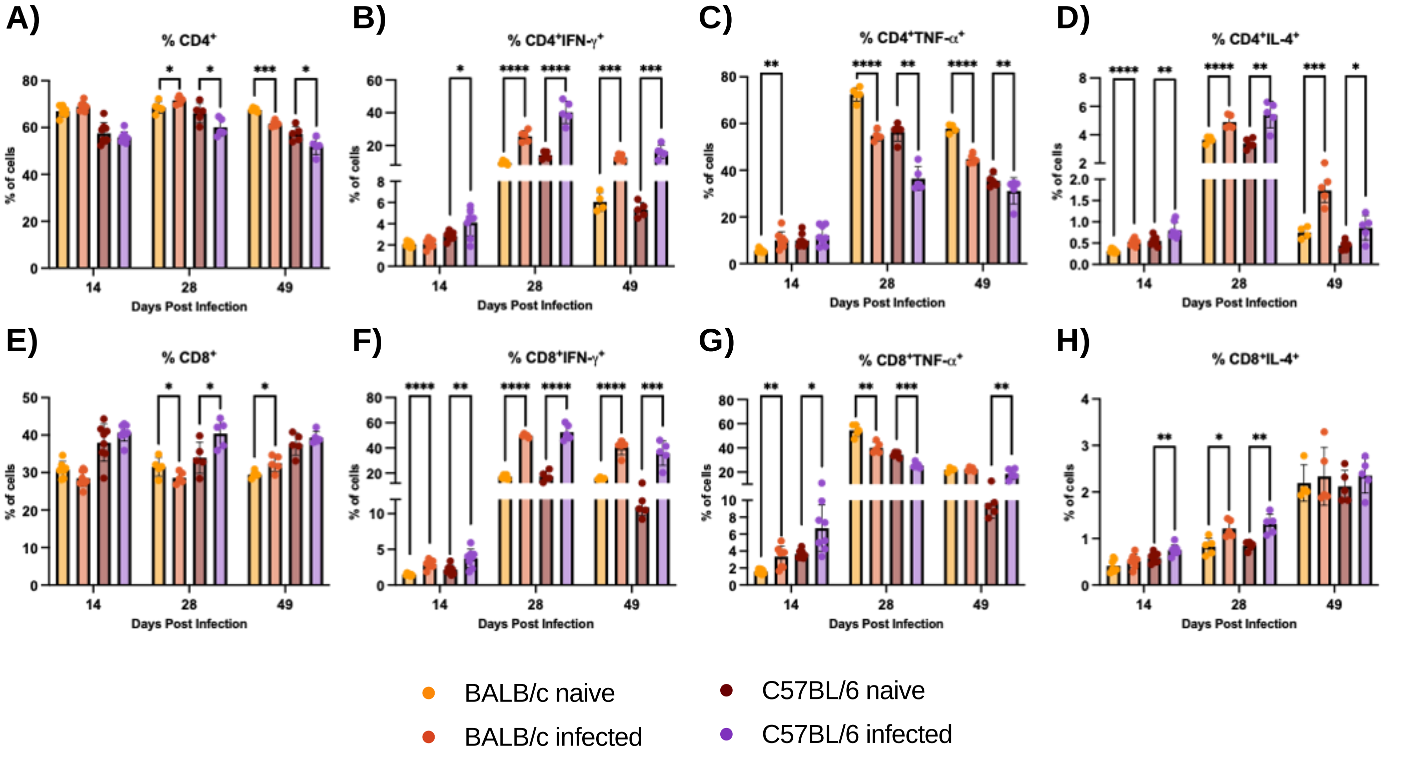

Figure S06: T cells response. Flow cytometry analysis of **A)** $CD3^+CD4^+$ **B)** $CD3^+CD4^+IFN\text{-}\gamma^+$ **C)** $CD3^+CD4^+TNF\text{-}\alpha^+$ **D)** $CD3^+CD4^+IL\text{-}4^+$ **E)** $CD3^+CD8^+$ **F)** $CD3^+CD8^+IFN\text{-}\gamma^+$ **G)** $CD3^+CD8^+TNF\text{-}\alpha^+$ and **H)** $CD3^+CD8^+IL\text{-}4^+$ T-cells. Each point represents an individual mouse: 14 DPI n = 8, 28 DPI n=5, and 49 DPI n=5. Significance was calculated by the Normality test followed by the parametric Unpaired t-test or nonparametric Mann-Whitney test. P-values of ≤0.05, ≤0.01, ≤0.001, and ≤0.0001 are represented as one, two, three, and four symbol characters, respectively.
